# Supplementary material for: Can Coagulation System Disorders and Cytokine and Inflammatory Marker Levels Predict the Temporary Clinical Deterioration or Improvement of Septic Patients on ICU Admission?
Source: J Clin Med. 2021 Apr 7;10(8):1548. doi: 10.3390/jcm10081548 (PMC8067680; doi:10.3390/jcm10081548)
Supplement: Supplementary file 1 [file jcm-10-01548-s001.pdf]

# Protein C SPSS (descriptive statistics)

```
SUMMARIZE
  /TABLES=ProteinC BY Prognosis
  /FORMAT=VALIDLIST NOCASENUM TOTAL LIMIT=100
  /TITLE='Case Summaries'
  /MISSING=VARIABLE
  /CELLS=COUNT MIN MAX MEDIAN.
```

## Summarize

**Notes**

| | | |
|---|---|---|
| Output Created | | 27-MAR-2021 04:21:48 |
| Comments | | |
| Input | Data | C:\Users\ΣΠΥΡΟΣ\Desktop\Sent to J Clin Med (Coagulopathy and Inflammation)\Coagulation etc in prognosis in sepsis 2.sav |
| | Active Dataset | DataSet1 |
| | Filter | <none> |
| | Weight | <none> |
| | Split File | <none> |
| | N of Rows in Working Data File | 28 |
| Missing Value Handling | Definition of Missing | For each dependent variable in a table, user-defined missing values for the dependent and all grouping variables are treated as missing. |
| | Cases Used | Cases used for each table have no missing values in any independent variable, and not all dependent variables have missing values. |

| | | |
|---|---|---|
| Syntax | | SUMMARIZE<br>/TABLES=ProteinC BY Prognosis<br>/FORMAT=VALIDLIST NOCASENUM TOTAL LIMIT=100<br>/TITLE='Case Summaries'<br>/MISSING=VARIABLE<br>/CELLS=COUNT MIN MAX MEDIAN. |
| Resources | Processor Time | 00:00:00,03 |
| | Elapsed Time | 00:00:00,02 |

```
[DataSet1] C:\Users\ΣΠΥΡΟΣ\Desktop\Sent to J Clin Med (Coagulopathy and
Inflammation)\Coagulation etc in prognosis in sepsis 2.sav
```

**Case Processing Summary[a]**

| | Cases | | | | | |
|---|---|---|---|---|---|---|
| | Included | | Excluded | | Total | |
| | N | Percent | N | Percent | N | Percent |
| ProteinC * 1=improvement,0=deterioration | 28 | 100,0% | 0 | 0,0% | 28 | 100,0% |

a. Limited to first 100 cases.

**Case Summaries[a]**

| | | | ProteinC |
|---|---|---|---|
| 1=improvement,0=deterioration | ,00 | 1 | 48,00 |
| | | 2 | 48,00 |
| | | 3 | 50,00 |
| | | 4 | 47,00 |
| | | 5 | 48,00 |
| | | 6 | 44,00 |
| | | 7 | 68,00 |
| | | 8 | 12,00 |
| | | 9 | 38,00 |

| | | | | |
|---|---|---|---|---|
| | | 10 | | 50,00 |
| | | 11 | | 46,00 |
| | | 12 | | 59,00 |
| | | 13 | | 54,00 |
| | | 14 | | 55,00 |
| | | 15 | | 36,00 |
| | | 16 | | 35,00 |
| | | 17 | | 29,00 |
| | | Total | N | 17 |
| | | | Minimum | 12,00 |
| | | | Maximum | 68,00 |
| | | | Median | 48,0000 |
| | 1,00 | 1 | | 48,00 |
| | | 2 | | 125,00 |
| | | 3 | | 48,00 |
| | | 4 | | 48,50 |
| | | 5 | | 68,00 |
| | | 6 | | 47,70 |
| | | 7 | | 84,00 |
| | | 8 | | 47,50 |
| | | 9 | | 63,00 |
| | | 10 | | 65,00 |
| | | 11 | | 82,00 |
| | | Total | N | 11 |
| | | | Minimum | 47,50 |
| | | | Maximum | 125,00 |
| | | | Median | 63,0000 |

**Case Summaries[a]**

| | | | ProteinC |
|---|---|---|---|
| 1=improvement,0=deterioration | Total | N | 28 |
| | | Minimum | 12,00 |
| | | Maximum | 125,00 |
| | | Median | 48,0000 |

a. Limited to first 100 cases.

# Protein C SPSS (ROC curve production)

```
ROC ProteinC BY Prognosis (1)
  /PLOT=CURVE(REFERENCE)
  /PRINT=SE COORDINATES
  /CRITERIA=CUTOFF(INCLUDE) TESTPOS(LARGE) DISTRIBUTION(FREE) CI(95)
  /MISSING=EXCLUDE.
```

## ROC Curve

**Notes**

| | | |
|---|---|---|
| Output Created | | 28-MAR-2021 02:25:03 |
| Comments | | |
| Input | Data | C:\Users\ΣΠΥΡΟΣ\Desktop\Sent to J Clin Med (Coagulopathy and Inflammation)\Revision 2\SPSS files\Coagulation etc in prognosis in sepsis 3.sav |
| | Active Dataset | DataSet1 |
| | Filter | <none> |
| | Weight | <none> |
| | Split File | <none> |
| | N of Rows in Working Data File | 28 |
| Missing Value Handling | Definition of Missing | User-defined missing values are treated as missing. |
| | Cases Used | Statistics are based on all cases with valid data for all variables in the analysis. |

| | | |
|---|---|---|
| Syntax | | ROC ProteinC BY Prognosis (1)<br><br>/PLOT=CURVE(REFERENCE)<br>/PRINT=SE COORDINATES<br><br>/CRITERIA=CUTOFF(INCLUDE) TESTPOS(LARGE) DISTRIBUTION(FREE) CI(95)<br>/MISSING=EXCLUDE. |
| Resources | Processor Time | 00:00:00,73 |
| | Elapsed Time | 00:00:00,72 |

```
[DataSet1] C:\Users\ΣΠΥΡΟΣ\Desktop\Sent to J Clin Med (Coagulopathy and
Inflammation)\Revision 2\SPSS files\Coagulation etc in prognosis in sepsis
3.sav
```

**Case Processing Summary**

| 1=improvement,0=deterioration | Valid N (listwise) |
|---|---|
| Positive[a] | 11 |
| Negative | 17 |

Larger values of the test result variable(s) indicate stronger evidence for a positive actual state.

a. The positive actual state is 1,00.

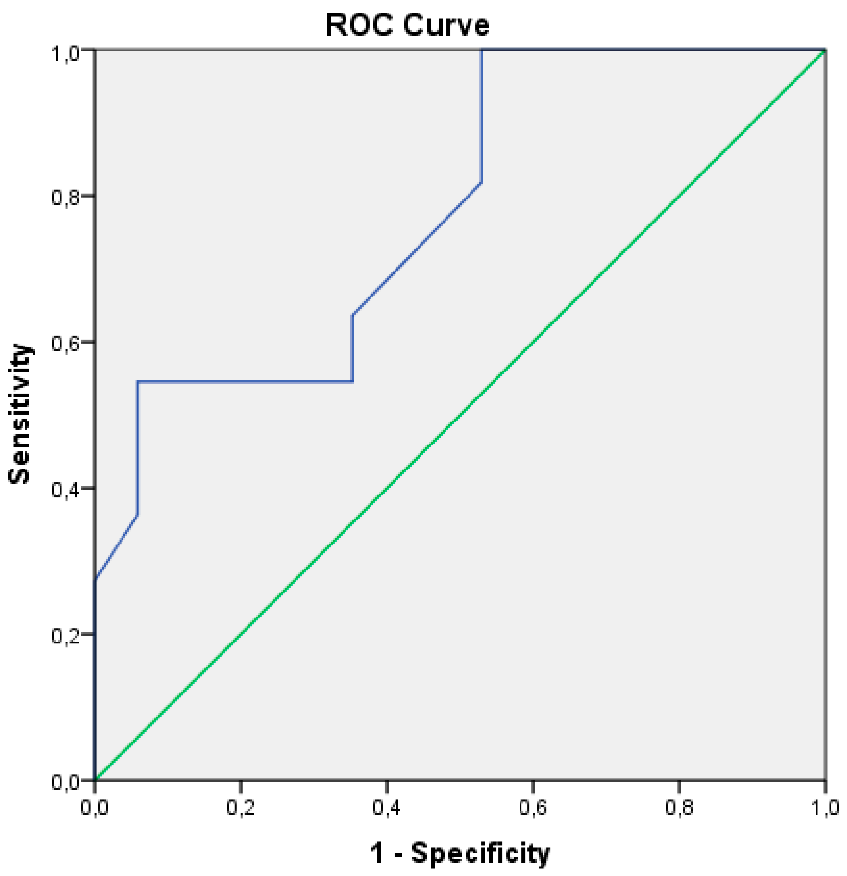

Diagonal segments are produced by ties.

**Area Under the Curve**

Test Result Variable(s): ProteinC

| Area | Std. Error[a] | Asymptotic Sig.[b] | Asymptotic 95% Confidence Interval | |
|---|---|---|---|---|
| | | | Lower Bound | Upper Bound |
| ,778 | ,089 | ,014 | ,603 | ,953 |

The test result variable(s): ProteinC has at least one tie between the positive actual state group and the negative actual state group. Statistics may be biased.

a. Under the nonparametric assumption

b. Null hypothesis: true area = 0.5

**Coordinates of the Curve**

Test Result Variable(s): ProteinC

| Positive if Greater Than or Equal To[a] | Sensitivity | 1 - Specificity |
|---|---|---|
| 11,0000 | 1,000 | 1,000 |
| 20,5000 | 1,000 | ,941 |
| 32,0000 | 1,000 | ,882 |
| 35,5000 | 1,000 | ,824 |
| 37,0000 | 1,000 | ,765 |
| 41,0000 | 1,000 | ,706 |
| 45,0000 | 1,000 | ,647 |
| 46,5000 | 1,000 | ,588 |
| 47,2500 | 1,000 | ,529 |
| 47,6000 | ,909 | ,529 |
| 47,8500 | ,818 | ,529 |
| 48,2500 | ,636 | ,353 |
| 49,2500 | ,545 | ,353 |
| 52,0000 | ,545 | ,235 |
| 54,5000 | ,545 | ,176 |
| 57,0000 | ,545 | ,118 |
| 61,0000 | ,545 | ,059 |
| 64,0000 | ,455 | ,059 |
| 66,5000 | ,364 | ,059 |
| 75,0000 | ,273 | ,000 |
| 83,0000 | ,182 | ,000 |
| 104,5000 | ,091 | ,000 |
| 126,0000 | ,000 | ,000 |

The test result variable(s): ProteinC has at least one tie between the positive actual state group and the negative actual state group.

a. The smallest cutoff value is the minimum observed test value minus 1, and the largest cutoff value is the maximum observed test value plus 1. All the other cutoff values are the averages of two consecutive ordered observed test values.
